# Mutation of a Single Residue Renders Human Tetherin Resistant to HIV-1 Vpu-Mediated Depletion

**DOI:** 10.1371/journal.ppat.1000443

**Published:** 2009-05-22

**Authors:** Ravindra K. Gupta, Stéphane Hué, Torsten Schaller, Ernst Verschoor, Deenan Pillay, Greg J. Towers

**Affiliations:** 1 Medical Research Council Centre for Medical Molecular Virology, Division of Infection and Immunity, University College London, London, United Kingdom; 2 Department of Virology, Biomedical Primate Research Centre, Rijswijk, The Netherlands; Northwestern University, United States of America

## Abstract

The recently identified restriction factor tetherin/BST-2/CD317 is an interferon-inducible trans-membrane protein that restricts HIV-1 particle release in the absence of the HIV-1 countermeasure viral protein U (Vpu). It is known that Tantalus monkey CV1 cells can be rendered non-permissive to HIV-1 release upon stimulation with type 1 interferon, despite the presence of Vpu, suggesting species-specific sensitivity of tetherin proteins to viral countermeasures such as Vpu. Here we demonstrate that Tantalus monkey tetherin restricts HIV-1 by nearly two orders of magnitude, but in contrast to human tetherin the Tantalus protein is insensitive to HIV-1 Vpu. We have investigated tetherin's sensitivity to Vpu using positive selection analyses, seeking evidence for evolutionary conflict between tetherin and viral countermeasures. We provide evidence that tetherin has undergone positive selection during primate evolution. Mutation of a single amino acid (showing evidence of positive selection) in the trans-membrane cap of human tetherin to that in Tantalus monkey (T45I) substantially impacts on sensitivity to HIV-1 Vpu, but not on antiviral activity. Finally, we provide evidence that cellular steady state levels of tetherin are substantially reduced by Vpu, and that the T45I mutation abrogates this effect. This study provides evidence that tetherin is important in protecting mammals against viral infection, and that the HIV-1 Vpu–mediated countermeasure is specifically adapted to act against human tetherin. It also emphasizes the power of selection analyses to illuminate the molecular details of host–virus interactions. This work suggests that tetherin binding agents might protect it from viral encoded countermeasures and thus make powerful antivirals.

## Author Summary

Pathogenic viruses have been infecting mammals throughout their evolution, exerting selective pressure to evolve systems to limit or eliminate these parasites. For example, intracellular proteins called restriction factors specifically restrict viral infection by targeting important viral processes. The restriction factor tetherin tethers newly formed HIV-1 virions to the surface of infected cells, preventing egress and further infection. In order to counteract tetherin, HIV-1 encodes a membrane-associated protein called Vpu that abrogates tetherin activity. Here we show that HIV-1 Vpu is inactive against tetherin from Tantalus monkeys and that this is due to a single amino acid that differs between human and tantalus monkey tetherin sequences. Evidence for positive selection at this position suggests that viral infections have provided the Darwinian selective pressure leading to this change. We also show that Vpu expression leads to a loss of tetherin protein in cells. Mutation of human tetherin protects it from HIV-1 Vpu activity, allowing functional protein expression and restriction of viral release. This study underlines the utility of selection analyses to reveal determinants of antiviral specificity and is strong evidence for the host–virus arms race described by the Red Queen hypothesis.

## Introduction

Retroviruses are obligate cellular parasites and as such rely on a wide variety of host proteins and pathways to complete their lifecycle. Moreover, they are subject to a variety of cellular antiviral activities that they must either overcome or avoid in order to successfully infect a cell. Together these positive and negative acting host factors combine to give primate lentiviruses narrow host ranges. For example, HIV-1 can only replicate in humans, chimpanzees and possibly gorillas [1]. A particular class of interferon inducible, cellular, innate immune factors, active against retroviruses, is referred to as restriction factors. These include TRIM5α [2], APOBEC3G (A3G), APOBEC3F (A3F) [3],[4] and tetherin/BST2/CD317 [5],[6]. Tetherin has been demonstrated to tether nascent retroviral virions to the plasma membrane, preventing their release from the infected cell. Instead, they are recruited back into the cell in endosomes for eventual destruction in the lysosome [5]–[8]. Tetherin has predicted trans-membrane and coiled coil regions as well as a predicted GPI anchor site [9]. It has also been shown to exist as a dimer and is glycosylated at two sites in its extracellular domain [10] although glycosylation does not appear to be important for restriction of Lassa or Marburg virus [11]. In a striking parallel to the antagonistic relationship between the antiviral A3G/F proteins and their HIV-1 encoded countermeasure Vif, the HIV-1 viral protein U (Vpu) counteracts the antiviral activity of tetherin [5],[6]. Vpu is a 16 kilodalton oligomeric type 1 trans-membrane protein encoded by an alternative reading frame in the env gene [12].

The antagonistic relationship between innate antiviral proteins, and the viruses that they restrict, is an excellent example of the Red Queen hypothesis [13]. This hypothesis proposes that pathogens and their hosts are locked in evolutionary conflict, each subject to selective pressure from the other to gain the advantage. This evolutionary arms race leads to alternate change followed by advantage and an overall maintenance of the relationship between host and pathogen. In support of this hypothesis, proteins such as TRIM5α and APOBEC3G have been shown to be under strong positive selection pressure throughout primate evolution, presumably from viruses that they target [14],[15]. Indeed, the study of adaptive selection and the analysis of species-specific restriction have illuminated details of the evolution of antiviral proteins as they change in response to rapidly evolving pathogens. Here we provide evidence for positive selection of tetherin and demonstrate that positively selected residues impact on sensitivity to Vpu but not on tetherin's anti HIV-1 activity. Furthermore, we show that mutation of a single residue renders human tetherin resistant to HIV-1 Vpu-mediated cellular depletion.

## Results

### Tantalus monkey tetherin restricts HIV-1 release but is not sensitive to HIV-1 Vpu

**Figure 1:**
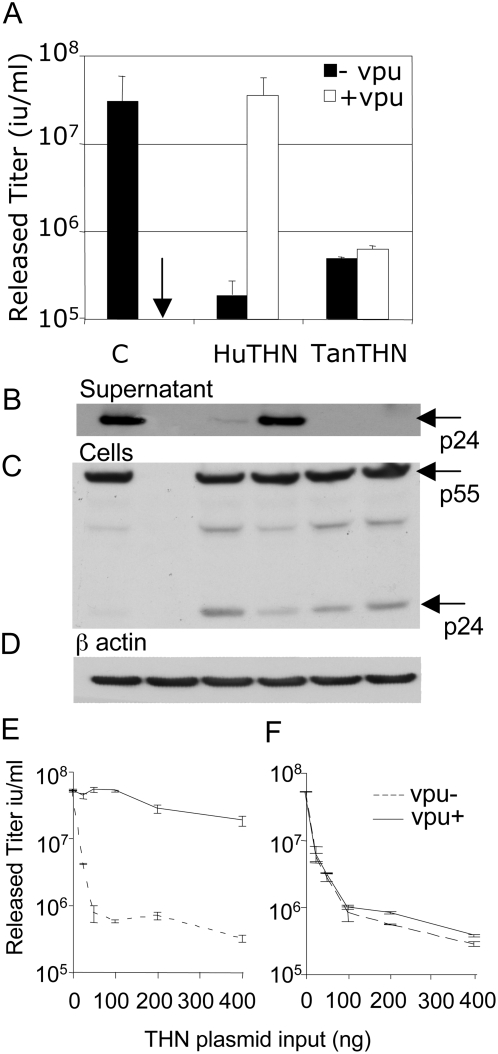
Tantalus monkey tetherin restricts HIV-1 release but is not sensitive to HIV-1 Vpu. (A) 293T cells were left untransfected (arrow) or co-transfected with HIV-1 Gag-pol (250 ng), HIV-1 YFP vector (375 ng), and VSV-G (250 ng) encoding plasmids alone (C) or along with plasmids encoding for human (HuTHN) or Tantalus monkey (TanTHN) tetherin (100 ng). Virus containing supernatants were titered on 293T cells and infectious titers were calculated. Human tetherin reduces HIV-1 release by 2 orders of magnitude and this is completely overcome by co-expression of HIV-1 Vpu. Tantalus monkey tetherin restricts HIV-1 release and is insensitive to HIV-1 Vpu. Error bars represent standard deviation of mean titers calculated from two independent experiments. HIV-1 Gag p24 and p55 bands are shown. (B) Measurement of HIV-1 p24 in the supernatant of transfected 293T cells by western blot using an anti p24 antibody demonstrates that p24 levels reflect infectious titers of the released virus (Figure 1A). (C) Measurement of HIV-1 Gag levels in extracts from transfected 293T cells demonstrates that tetherin expression does not reduce viral protein expression. (D) Cell extract blots in C were stripped and re-probed for β actin as a loading control. (E) 293T cells were co-transfected with HIV-1 vector plasmids, 200 ng Vpu plasmid and a titration of human tetherin plasmid (E) or Tantalus monkey tetherin plasmid (F) as shown. Titration of human tetherin demonstrates that Vpu counteracts human tetherin at high and low tetherin doses whereas titration of Tantalus monkey tetherin demonstrates that Vpu does not counteract Tantalus tetherin even when tetherin doses are low.

To measure tetherin antiviral activity and its abrogation by the viral Vpu protein, we utilised HIV-1 YFP encoding vectors. Transfection of 293T cells with HIV-1 vector plasmids leads to production and release of HIV-1 virions containing a YFP encoding genome. The efficiency of virion release can then be measured by titration of the 293T supernatant on permissive target cells. Expression of viral proteins and release of virions from transfected cells was also assayed by western blot, detecting HIV-1 capsid in extracts from the transfected cells and the supernatant respectively. Expression of HIV-1 vector plasmids in the absence of tetherin or Vpu led to production of HIV-1 YFP with a titer of around 5×10^7^ infectious units/ml. Co-expression of human tetherin significantly reduced infectious HIV-1 vector production and co-expression of HIV-1 Vpu completely rescued HIV-1 YFP release (Figure 1A). These data, demonstrating restriction of HIV-1 by human tetherin, and the ability of HIV-1 Vpu to act as countermeasure to tetherin, are concordant with described observations [5],[6].

Tantalus monkey CV1 cells are able to release HIV-1 in a Vpu insensitive way [8]. Furthermore, after interferon treatment these cells support reduced HIV-1 release. We hypothesised that this might be due to interferon induced expression of a tetherin protein that was insensitive to HIV-1 Vpu. To test this we cloned the Tantalus monkey tetherin from CV1 cells and co-expressed it with HIV-1 vector plasmids as above. Indeed, expression of the Tantalus tetherin protein restricted HIV-1 YFP release, by almost 2 orders of magnitude (Figure 1A). Importantly, and in concordance with HIV-1 Vpu's inability to stimulate HIV-1 release from CV1 cells [8], restriction by Tantalus tetherin was insensitive to co-expression of HIV-1 Vpu. This observation suggests that the Vpu mediated tetherin countermeasure is species-specific and that the HIV-1 Vpu protein cannot counteract the antiviral activity of the Tantalus tetherin protein. Measurement of Gag levels in the supernatant and transfected cells by western blot with a p24 CA antibody demonstrated that viral titers (Figure 1A) reflect the amount of p24 released into the supernatant (Figure 1B) and that tetherin expression did not impact on Gag expression levels (Figure 1C). β actin was measured as a loading control (Figure 1D). We note that there is evidence for increased levels of protease cleaved Gag in the cell extracts in the presence of tetherin restriction, consistent with the notion that maturing particles are tethered to the surface of the restrictive cells.

It is possible that Tantalus tetherin is insensitive to Vpu because it is expressed more efficiently than the human protein and it therefore saturates the HIV-1 Vpu protein. To test this we titrated both human (Figure 1E) and Tantalus tetherin (Figure 1F) against a fixed dose of Vpu and measured the titer of the released virus. In fact human tetherin was counteracted by Vpu at high or low doses whereas Tantalus tetherin was not significantly counteracted by HIV-1 Vpu, even when the dose of tetherin was low. These data are consistent with the notion that Tantalus tetherin is insensitive to the HIV-1 encoded tetherin countermeasure Vpu.

### Selection analyses reveal positively selected tetherin residues in the primate lineage

**Figure 2:**
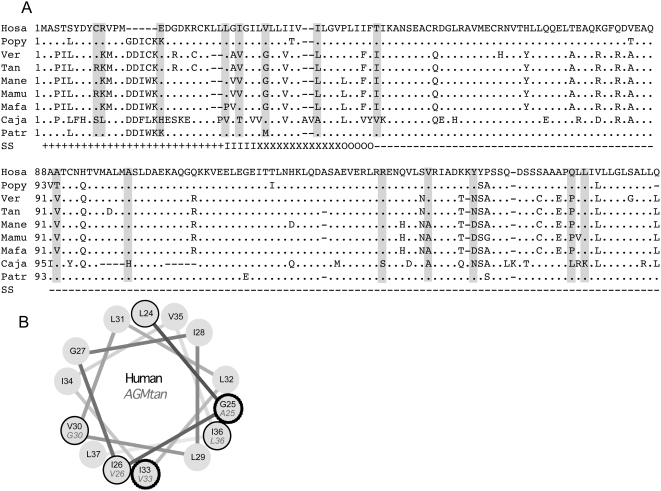
Selection analyses reveal positively selected tetherin residues in the primate lineage. (A) Nucleotide alignment of nine primate tetherin sequences. HoSa (Homo Sapiens), Popy (Pongo Pygmaeus (Orangutan)) Patr (Pan Trolodytes (Chimpanzee)), Tan (Tantalus monkey), Ver (Vervet monkey), Mane (Macaque Nemestrina (pigtailed macaque)), Mafa (Macaque Fasicularis (cynomolgus monkey)), Mamu (Macaque Mulatta (Rhesus macaque)) Caja (Callithrix jacchus (white tufted ear marmoset). Codon positions under positive selection are indicated by shaded boxes. Secondary structure (SS) was predicted by PSIPRED [53] and is symbolised as (+) cytoplasmic domain; (I) trans-membrane domain inner cap; (X) trans-membrane domain alpha helix; (O) trans-membrane domain outer cap; (−) extra-cellular domain. (B) Predicted structure of the trans-membrane helix performed using helical wheel projection (http://rzlab.ucr.edu/scripts/wheel/wheel.cgi) suggests that residues 26, 30, and 36 are on the same side of the protein. Closed circles indicate positively selected amino acids, dashed circles indicate residues that are different between human and Tantalus monkey, but not positively selected. Human amino acids are shown in black and Tantalus monkey in grey italic.

Species specificity of the tetherin/Vpu interaction is reminiscent of the species specificity of HIV-1 Vif activity against primate APOBEC3G proteins, as well as the species specificity of TRIM5α against retroviruses. In both of these examples the determinants of specificity can be revealed by analysis of positive selection in the species-specific variants of each restriction factor [14]–[17]. We therefore gathered tetherin sequences from a variety of primates and aligned them to the Tantalus monkey tetherin sequence (Figure 2A). The alignment revealed that primate tetherin sequences are divergent (mean pairwise genetic difference of 0.116 nucleotide substitutions per site, standard deviation 0.085 substitutions/sites), yet 93 out of 180 amino acid sites are conserved along the primate alignment, excluding positions with gaps.

We examined the alignment of primate tetherin sequences for evidence of heterogeneity of synonymous (*d_N_*) and non-synonymous (*d_S_*) substitution rates, indicative of adaptive selection. An excess of non-synonymous substitutions, which lead to protein sequence change, compared to synonymous substitutions, which do not, (*d_N_/d_S_*>1) is traditionally regarded as indicative of positive (or adaptive) selection. Conversely, a *d_N_/d_S_*<1 suggests negative (or purifying) selection. Although the average rates of synonymous changes exceeded rates of non-synonymous changes across the sequence alignment, reflecting a predominance of purifying selection on the tetherin genes (average *d_N_/d_S_* = 0.93; 95% Confidence Intervals = 0.76; 1.11), evidence for positive selection was found when maximum likelihood models allowing variable *d_N_/d_S_* ratios among sites were applied to the data. The model allowing sites to evolve under positive selection had a significantly better fit to the data than the model assuming no positive selection (likelihood ratio test with 2 degrees of freedom; p = 0.018). Furthermore, analyses of codon-specific positive selection in the primate lineage revealed fifteen residues potentially under adaptive selection, five of which, positions 24, 26, 30, 36 and 45 in the human tetherin sequence, were found in or bordering the predicted trans-membrane domain (Figure 2A, Table 1). We note that the three selected trans-membrane residues present in the central helix are predicted to be on the same side of the helix and therefore in close proximity to one another (Figure 2B). Additional sites showing evidence of positive selection were found in the predicted cytoplasmic and C terminal extra-cellular domains (Table 1).

**Table 1:** Positive selection in the tetherin gene.

Codon*	Mean dS	Mean dN	P(dN>dS)**	Bayes Factor
9	0.844	2.056	0.959	39.20
10	0.802	2.055	0.969	51.30
14	0.832	2.066	0.967	49.22
**24**	0.771	2.004	0.949	30.73
**26**	0.789	2.057	0.972	57.62
**30**	0.805	2.004	0.941	26.65
**36**	0.747	2.059	0.983	95.47
**45**	0.800	2.007	0.944	28.01
89	0.772	1.985	0.938	25.38
100	0.746	1.996	0.950	31.59
139	0.763	1.992	0.944	28.05
146	0.802	1.991	0.935	24.03
153	0.873	2.005	0.927	21.14
167	0.817	1.994	0.933	23.30
169	0.790	2.011	0.948	30.31

Codon-specific rates of synonymous (*d_S_*) and non-synonymous (*d_N_*) nucleotide substitutions were estimated by Random Effect Likelihood methods under the MG94xHKY85 model of evolution. The Bayesian posterior probability for positive selection is given for each codon position. A Bayes factor of greater than 20 at a given site was considered to be strong support for positive selection.

*: Codon positions according to the human tetherin sequence (NM004335). Codons in the trans-membrane region are indicated in bold.

**: Posterior probability for positive selection (dN>dS) at the site.

### The positively selected tetherin trans-membrane region residues impact on sensitivity to HIV-1 Vpu

**Figure 3:**
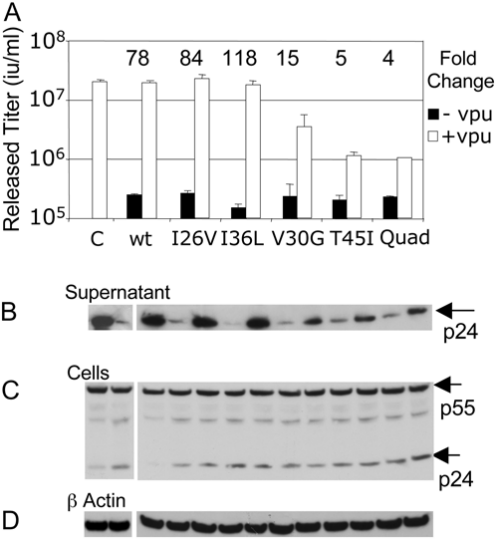
The positively selected tetherin trans-membrane region residues impact on sensitivity to HIV-1 Vpu. (A) Co-expression of HIV-1 plasmids alone (C) with wild-type human tetherin (WT) and measurement of HIV-1 release in the absence of HIV-1 Vpu (white bar) or presence (black bar). Mutation of positively selected residues I26V, V30G, I36L, T45I (Quad) in the trans-membrane region of human tetherin results in reduced sensitivity to Vpu whilst maintaining similar antiviral activity. The effect of single mutations are also shown. Errors are standard error of the mean of 2 experiments. Equal amounts of tetherin plasmids were used (100 ng) (B) Measurement of HIV-1 p24 in the supernatant of transfected 293T cells by western blot (C) Measurement of HIV-1 Gag levels in extracts from transfected 293T cells. (D) Cell extract blots in C were stripped and re-probed for β actin as a loading control.

Knowing that HIV-1 Vpu counteracts human tetherin we hypothesised that the differences between primate tetherin sequences might be due to selective pressure from pathogenic retroviruses encoding tetherin countermeasures. We focused on the trans-membrane domain as a likely site for Vpu interaction because both tetherin and Vpu are integral membrane proteins and replacing the Vpu trans-membrane domain with that from CD8 causes mislocalisation and loss of Vpu activity [7]. Furthermore, a non-functional Vpu with a scrambled trans-membrane region co-localised less extensively with tetherin [6]. We hypothesised that changing the trans-membrane region residues in the human tetherin protein to those in the Tantalus monkey protein should impact on sensitivity to HIV-1 Vpu. Of positions 24, 26, 30, 36 and 45, which are in or bordering the predicted trans-membrane region (Figure 2), position 24 is conserved between human and Tantalus monkey. We therefore made a human quadruple tetherin mutant I26V, V30G, I36L, T45I and tested its antiviral activity and sensitivity to HIV-1 Vpu. The wild-type human tetherin suppressed HIV-1 release reducing infectious titer by 78 fold and release was completely rescued by HIV-1 Vpu expression (Figure 3A). Conversely, the human tetherin quadruple mutant (Quad) was able to potently suppress HIV-1 release but was only weakly rescued by co-expression of HIV-1 Vpu, 4 fold vs 78 fold rescue for the wild-type protein (Figure 3A–3D). Importantly, the mutations did not significantly reduce tetherin's antiviral activity on HIV-1 release (Figure 3A, black bars). In order to examine the contribution of each selected residue to Vpu sensitivity we tested single mutants for antiviral activity and Vpu sensitivity. In fact, mutating human residue 30 (V30G) moderately reduced its Vpu sensitivity (from 78 to 15 fold) whereas mutating residue 45 (T45I) had a similar impact as mutating all 4 residues (5 fold versus 4 fold rescue on Vpu expression). Remarkably, it appears that human tetherin can escape the HIV-1 encoded tetherin countermeasure Vpu and restrict HIV-1 if a single tetherin amino acid is changed to reflect the Tantalus monkey sequence. Indeed, the evidence for positive selection suggests that the tetherin gene has been under pressure to change at this position during primate evolution.

For all of the experiments in Figure 3A we measured Gag levels by western blot in the cells and p24 levels in the supernatant (Figure 3B–3D). In each case p24 levels in the supernatant reflected the titer of the virus as plotted (Figure 3B) Furthermore, Gag expression levels were similar in the cells and unaffected by tetherin expression (Figure 3C). β actin levels in cell lysates were measured as a loading control (Figure 3D).

### HIV-1 Vpu expression leads to a loss of wild-type but not mutant tetherin proteins

**Figure 4:**
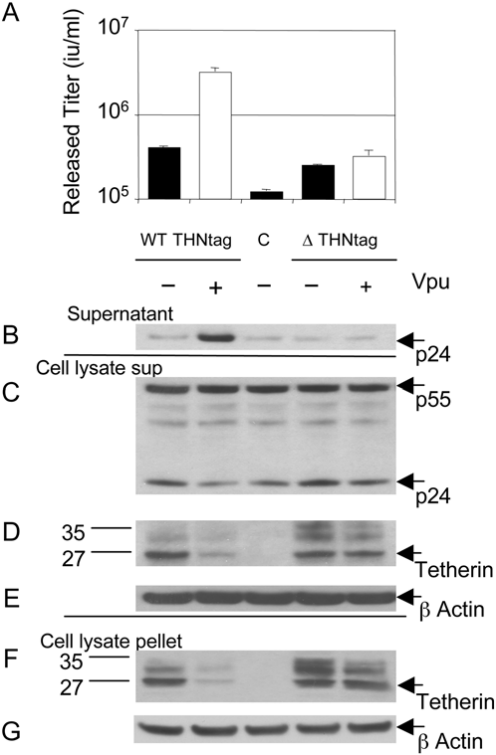
Vpu expression leads to a loss of wild-type but not a quadruple mutant tetherin protein steady state levels. (A) Co-expression of HIV-1 plasmids and wild-type (WT) N terminally tagged human tetherin and measurement of HIV-1 release in the absence of HIV-1 Vpu (black bar), or presence (white bar). Mutation of positively selected residues I26V, V30G, I36L, T45I (ΔTHN) in the trans-membrane region of human tetherin results in reduced sensitivity to Vpu whilst maintaining antiviral activity. The effect of co-transfection of HIV-1 plasmids and untagged human tetherin is shown for comparison (Lane C). The titre of the unrestricted HIV-1 was 10^7^ infectious units/ml. Errors are standard error of the mean of 2 experiments. (B) Measurement of HIV-1 p24 in the supernatant of transfected 293T cells by western blot (C) Measurement of HIV-1 Gag levels in extracts from transfected 293T cells. Cell extract lysates (D) or pellets (F) were blotted for the Xpress tag to detect tetherin. Sizes of molecular weight markers are shown in kilodaltons. Blots in D (E) or F (G) were stripped and re-probed for β actin as a loading control. Data are representative of 3 independent experiments and similar results were seen with an N terminal HA tag.

**Figure 5:**
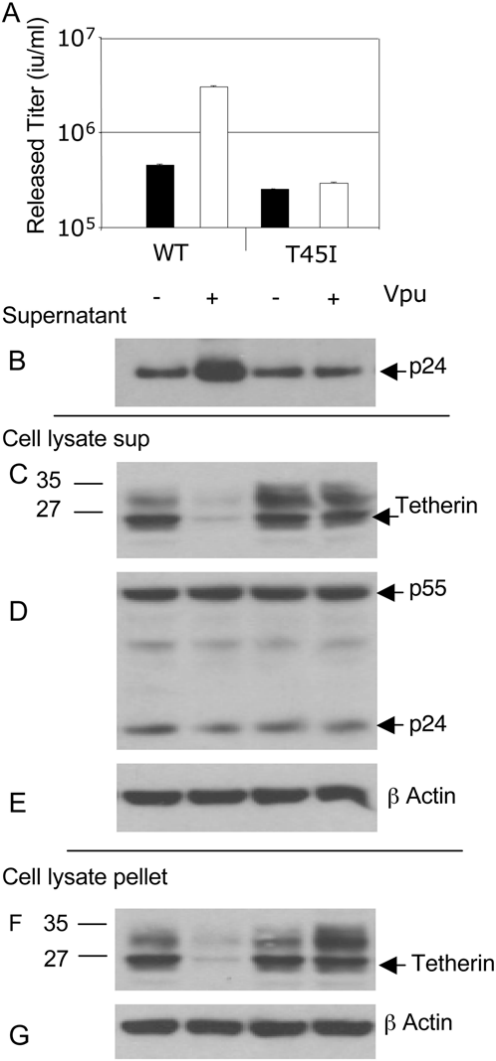
Mutation of a single amino acid (T45I) leads to insensitivity to Vpu and persistence of tetherin protein. (A) Co-expression of HIV-1 plasmids and wild-type (WT) N terminally tagged human tetherin and measurement of HIV-1 release in the absence of HIV-1 Vpu (black bar), or presence (white bar). Mutation T45I in the trans-membrane region of human tetherin results in insensitivity to Vpu whilst maintaining antiviral activity. The titre of the unrestricted HIV-1 was 10^7^ infectious units/ml. Errors are standard error of the mean of 2 experiments. (B) Measurement of HIV-1 p24 in the supernatant of transfected 293T cells by western blot (C) Measurement of HIV-1 Gag levels in cleared RIPA extracts from transfected 293T cells. Tetherin was detected by western blot of N-terminal Xpress tag in the cleared RIPA extract supernatants (D) and pellets (F) as shown. Sizes of molecular weight markers are shown in kilodaltons. Blots in (E) and (G) have been stripped and re-probed for β actin as a loading control. Data are representative of 2 independent experiments.

It is formally possible that the tetherin mutations responsible for reduced sensitivity to Vpu impacted on its expression levels. In order to control for this possibility, and in the absence of a tetherin antibody, we appended an N terminal epitope tag to the wild-type and quadruple mutant human tetherin proteins and performed an assay for tetherin function and Vpu sensitivity, as above. Surprisingly, we found that the tag slightly reduced human tetherin's antiviral activity, compare Figure 4A to Figure 3A. Assay of p24 in supernatant (Figure 4B) and cell lysate (Figure 4C) confirmed this observation. Nonetheless, we measured the expression levels of the tagged tetherin proteins by western blot, reasoning that if expression levels were changed by the four mutations then this would be evident in expression levels of the tagged proteins, despite their reduced activity. In fact, the wild-type and mutant unglycosylated proteins were expressed at similar levels in the absence of Vpu (Figure 4D). Importantly, co-transfection of Vpu led to reduction in the amount of tetherin detected, both in a cleared RIPA cell lysate supernatant and the associated pellet as described [18] (Figure 4D and 4F). β actin was measured as a loading control in both supernatant and pellet (Figure 4E and 4G). These observations are concordant with those reported by Bartee et al who reported lower levels of tetherin protein in the presence of Vpu [19]. We obtained similar results when the experiment was carried out using the human single point mutant of tetherin T45I demonstrating that this single mutation can render tetherin insensitive to Vpu (Figure 5). These experiments suggest that the steady state level of tetherin is reduced by co-expression of HIV-1 Vpu, and that mutation at positively selected sites leads to a persistence of the tetherin protein presumably due to impaired interaction with the tetherin trans-membrane region.

### HIV-1 Vpu mediated loss of tetherin is partially rescued by MG132

**Figure 6:**
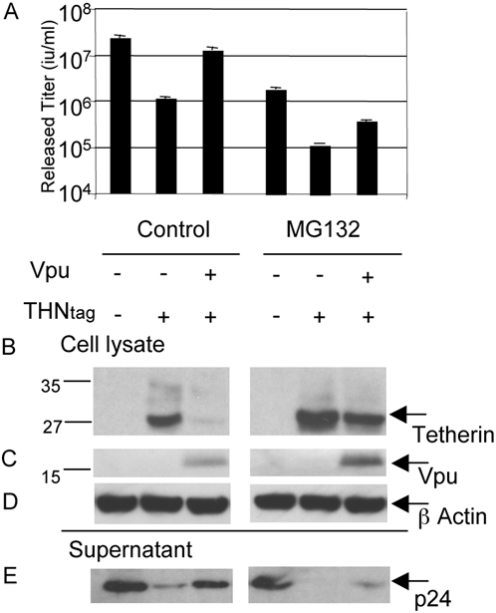
HIV-1 Vpu–mediated loss of tetherin is partially abrogated by MG132. (A) Co-expression of HIV-1 plasmids and wild-type (WT) N terminally tagged human tetherin and measurement of HIV-1 release in the presence or absence of HIV-1 Vpu-HA co-expression and proteasome inhibitor MG132 (0.8 µM for 12 hours) as shown. Errors are standard error of the mean of 2 experiments. (B) Tetherin was detected by western blot of Xpress tag in cleared RIPA extract supernatants and pellets as shown. Sizes of molecular weight markers are shown in kilodaltons. (C) Vpu-HA was detected in sonicated RIPA extract as shown (D) Blots in B have been stripped and re-probed for β actin as a loading control. (E) Measurement of HIV-1 p24 in the supernatant of transfected cells by western blot. Data are representative of 2 independent experiments.

Next we considered whether inhibition of the proteasome impacts on the Vpu mediated reduction of tetherin steady state levels. We co-expressed HIV-1 vectors and N-terminally tagged wild-type human tetherin, and examined the impact of HIV-1 Vpu-HA co-expression in the presence and absence of the proteasome inhibitor MG132 (Figure 6). Consistent with previous observations, MG132 lowered infectious titres, a phenomenon attributed to depletion of ubiquitin pools required for HIV-1 maturation and release [20] (Figure 6A). In addition, MG132 increased cellular levels of tetherin in the absence of Vpu, consistent with the notion that tetherin (like many other cellular proteins) is cycled within the host cell using ubiquitin dependent pathways. MG132 also significantly increased levels of Vpu (Figure 6C, compare left and right panels), consistent with previous observations [21].

Vpu leads to a loss in tetherin expression levels and a rescue of HIV-1 titre in the supernatant (Figure 4 and Figure 5). However, treatment with the proteasome inhibitor MG132 reversed the depletion of tetherin levels induced by Vpu. The drug reduced tetherin antagonism by Vpu although tetherin continued to partially inhibit the release of HIV-1, despite Vpu's presence (Figure 6B, compare left and right panels). This observation suggests that the proteasome is involved in Vpu mediated reduction of tetherin levels. Moreover, Vpu's ability to partially rescue HIV-1 release, despite being inhibited for tetherin degradation, suggests that it can inhibit tetherin via sequestration or mislocalisation. Indeed, Vpu has been demonstrated to reduce tetherin's cell surface expression and MG132 treatment did not completely restore it to the surface levels seen in the absence of Vpu, again suggesting sequestration or mislocalisation of tetherin by Vpu [6]. Since inhibiting the proteasome impacts on the levels of free ubiquitin it is also possible that tetherin is degraded by a proteasome independent pathway such as trafficking to lysosomes via endosomal sorting pathways, which are known to depend on ubiquitination [22]. We also note that MG132 treatment increases tetherin levels, raising the possibility of Vpu saturation. However, the fact that we still see maximal Vpu activity when the Tetherin plasmid dose is increased from 100 to 400 nanograms (Figure 1), as well as increased levels of Vpu (Figure 6), suggests that increased levels of tetherin protein are unlikely to explain the inhibition of Vpu mediated tetherin depletion by MG132 treatment.

## Discussion

Here we provide evidence that the tetherin/CD317/BST-2 host restriction factor has been subject to positive selection during mammalian evolution. We hypothesised that the selected changes might impact on sensitivity to viral encoded tetherin countermeasures and in support of this human tetherin becomes largely insensitive to the HIV-1 encoded countermeasure, the Vpu protein, when it is mutated to represent the Tantalus monkey sequence at a single position (T45I). A second positively selected residue in the trans-membrane region V30 also impacts on sensitivity to HIV-1 Vpu, although less dramatically than T45I, when mutated to glycine. We also show that the single point mutant T45I is able to render human tetherin resistant to Vpu-mediated cellular depletion, and furthermore that the mechanism involves the proteasome or a ubiquitin-dependent pathway. This is consistent with the observation that Vpu recruits CD4 to the βTrCP subunit of the SCF(βTrCP) ubiquitin ligase complex leading to degradation via the proteasome [23]. Concordantly, Goffinet and colleagues have recently reported that HIV-1 Vpu mediates proteasomal degradation of human but not rodent tetherin proteins and that this is abrogated by inhibition of the proteasome with ALLN or clasto-lactacysteine [24].

In the final stages of preparation of this manuscript, McNatt and colleagues reported the findings of a similar study on species-specificity of tetherin's responsiveness to HIV-1 Vpu [25]. In contrast to our approach these investigators used chimeric constructs to show that the TM region conferred sensitivity to HIV-1 Vpu, before locating specific sensitivity determinants using systematic mutagenesis. Subsequent positive selection analysis was consistent with our findings concluding that tetherin has been under positive selection in primates.

The observation that a single mutation (T45I) can render tetherin largely insensitive to HIV-1 Vpu mediated degradation is reminiscent of point mutations impacting on APOBEC3G's sensitivity to HIV-1 Vif [26] as well as point mutations in either capsid [27],[28], TRIM5 [29], TRIMCyp [30], or Fv1 [31],[32], which strongly impact on sensitivity to restriction. Indeed, it appears to be a common theme of the interaction between restriction factors and viral proteins that only one or two amino acids dictate the difference between replication and restricted infection.

In this study we have focused on the trans-membrane domain of tetherin. It seems likely that other tetherin sensitive viruses and their countermeasures may have caused selection at the positions outside of the trans-membrane domain. Vpu has been shown to facilitate the release of distantly related viruses including the gamma retrovirus murine leukaemia virus, as well as the sheep lentivirus maedi-visna virus [5],[33]. Vpu has also been shown to improve release of VLPs derived from the filovirus ebola [8] and Marburg and Lassa viruses [11]. These viruses do not appear to encode Vpu homologues and it is unclear whether they encode tetherin countermeasures. However, there is evidence that certain viruses have tetherin countermeasures unrelated to HIV-1 Vpu. For example, Kaposi's sarcoma associated herpes virus (KSHV) encodes a protein K5, known to reduce tetherin cell surface expression [19]. Moreover, some primate lentiviruses, such as HIV-2, are thought to have anti-tetherin function mediated by their envelope protein [34]–[36]. Moreover, Ebola virus glycoprotein has recently been shown to counteract tetherin antiviral activity [37]. We therefore speculate that viruses with countermeasures unrelated to HIV-1 Vpu are responsible for the positive selection of tetherin outside of the trans-membrane region.

Our observations are evidence for a dynamic evolutionary arms race, as described by the Red Queen hypothesis, between tetherin and virus encoded countermeasures such as Vpu. They are strong evidence for tetherin having a critical role in innate immunity against retroviral infection throughout mammalian evolutionary history and underline the utility of seeking evidence for positive selection to reveal details of host virus interactions. The details of the antiviral mechanism of tetherin have been partially uncovered. Tetherin restricted viruses are prevented from leaving the surface of infected cells and are subsequently endocytosed in a Rab5a dependent way [5]–[8]. The restricted viruses achieve a very late stage of viral budding and can be released by proteolytic cleavage from infected cells [5],[7]. Vpu appears to counteract tetherin by sequestering it from the cell surface [5],[6] and our data support recent findings that Vpu causes tetherin degradation via the proteasome. This observation suggests that Vpu may work in the same way as Vif and act as an adapter protein that recruits tetherin to be degraded [38],[39].

Future work will include identification of countermeasures from other viruses, which are likely to have independent mechanisms for antagonising tetherin. The potential for translational application of these findings is substantial. Identification of inhibitors for Vpu, or indeed other virus-encoded countermeasures, could have powerful therapeutic potential. The multifunctional nature of Vpu, for example its ability to reduce CD4 surface expression [40], will presumably improve potency of Vpu inhibition. We also envisage tetherin binding drugs that protect it from multiple viral encoded counter measures and are therefore broadly active against different classes of enveloped viruses.

## Materials and Methods

### Sequences and cloning

Primate tetherin sequences were retrieved using BLAST [41] and manually aligned. Sequences used were *Homo sapiens* human (NM004335), *Pan troglodytes* chimpanzee (XM_512491), *Macaca fascicularis* cynomolgus macaque (CJ479048), *Macaca nemestrina* pigtailed macaque (DY743778), *Macaca mulatta* rhesus macaque (CB554098), *Chlorocebus pygerythrus* vervet monkey. Tetherin sequences from orangutan and marmoset were inferred using BLAT (http://genome-mirror.duhs.duke.edu/cgi-bin/hgBlat) on the *Pongo pygmaeus abelii* orangutan genome and *Callithrix jacchus* marmoset genome. Orangutan sequence was confirmed by PCR cloning individual exons and sequencing.

Tantalus monkey and pig tetherin cDNAs were PCR cloned from the *Chlorocebus tantalus* (Tantalus monkey) CV1 cell line or the porcine cell line ST IOWA respectively, as described [42] using Tantalus monkey primers Fwd 5′ - CGATGCGGCCGCCCACCATGGCACCTATTTTGTATG Rev 5′ – GCCGATCTCGAGTCACAGCAGCAGAGCGCTCAAGC and pig primers Fwd 5′-ATGTCACCTAGTTTGTATTCC-3′ and Rev 5′-ACACCTCAGGTCAGCAG-3′ and inserted into pcDNA3.1 (Clontech). Cv1 cells are assumed to be derived from Tantalus monkey due to characteristic polymorphism in the CCR5 gene [43]. Four independent clones of each cDNA were sequenced. Tetherin sequences have accession numbers FJ345303 Tantalus monkey and FJ527910 pig. Site directed mutagenesis was performed using QuikChange (Stratagene). Human wild-type and mutant tetherin proteins were epitope tagged by cloning into the pCDNA4 vector encoding an N terminal Xpress epitope tag (Invitrogen) between the Not1 and Xho1 sites.

### Phylogenetic and selection analyses

Pairwise genetic distances between the nine primate tetherin sequences were calculated under the General Time Reversible model of nucleotide substitutions with proportion of invariable sites and gamma-distributed rate heterogeneity, using the program Paup* [44].

Evidence for positive selection in the tetherin gene along the primate lineage was sought by comparison of synonymous (*d_S_*) and non-synonymous (*d_N_*) substitution rates using the program codeML from the PAML package [45] and the Random Effect Likelihood (REL) [46] method implemented by the Datamonkey web-based facility [47].

An excess of non-synonymous substitutions compared to synonymous substitutions (i.e. *d_N_/d_S_*>1) is thought to be indicative of positive (or diversifying) selection, whereas *d_N_/d_S_*<1 suggests negative (or purifying) selection.

In codeML, the sequence alignment and a corresponding neighbor-joining phylogeny were successively submitted to a model in which sites are distributed into categories where *d_N_*/*d_S_* is beta-distributed between 0 and 1 (M7) and to a model in which sites are distributed into categories where *d_N_*/*d_S_* is beta-distributed between 0 and 1, with an extra category where *d_N_*/*d_S_* is freely estimated (M8). A significant better fit of M8 than M7, as indicated by a likelihood ratio test with 2 degrees of freedom, was taken as an evidence of positive selection.

The REL algorithm was used to identify potential codon positions evolving under positive selection. After estimating branch lengths and substitution rates under the Hasegawa-Kishino-Yano (HKY85) model of evolution, the MG94xHKY85 codon model [48] was fitted to the data to obtain independent rate distributions for *d_N_* and *d_S_*. For each codon, Bayes Factors for the events that *d_N_*<*d_S_* (indicative of negative selection) and that *d_N_*>*d_S_* (indicative of positive selection) at that site were estimated. A Bayes Factor of 20 or more in favor of *d_N_*>*d_S_* was considered strong support for adaptive selection at that site.

### Viral infection assays

Preparation of VSV-G pseudotyped, YFP encoding HIV-1 has been described [49]. Briefly 10^6^ 293T cells per well were cotransfected in six well plates using 6 µl Fugene-6 (Roche) with the *gag-pol* expression vector p8.91 (250 ng) [50], pMDG encoding the Vesticular Stomatis Virus G glycoprotein (VSV-G) (250 ng) [51] and HIV-1 vector encoding YFP (375 ng) [52]. 100 ng of tetherin constructs were co-transfected along with either 200 ng of HIV-1 Vpu or empty vector (pCDNA3.1, Invitrogen). After 48 hours the supernatant was harvested, filtered and titered on 293T cells as described [49].

### Western blots

HIV-1 p24 was measured in supernatants or cell pellets by western blot as described [18] using HIV-1 p24 monoclonal antibody (183-H12-5C), a gift from the NIH AIDS Research and Reference Reagent Programme. Membranes were then stripped and reprobed for β actin as a loading control. Tetherin extracts were made by lysing cells in RIPA buffer. Cleared lysate was added to laemmli buffer and the pellet was solubilised in laemmli buffer by sonication. Samples were then boiled before separation by SDS PAGE, all as previously described [18]. Xpress epitope tag was detected using mouse anti-Xpress antibody (Invitrogen). HA epitope tag was detected using mouse anti-HA antibody (Covance).
